# Examining the Indirect Death Surveillance System of The Great East Japan Earthquake and Tsunami

**DOI:** 10.3390/ijerph191912351

**Published:** 2022-09-28

**Authors:** Xiang Zheng, Chuyao Feng, Mikio Ishiwatari

**Affiliations:** 1 Department of International Studies, University of Tokyo, Chiba 277-8561, Japan; 2 Japan International Cooperation Agency, Tokyo 151-0066, Japan

**Keywords:** indirect death, long-term effects, excess mortality, earthquake fatalities, surveillance system

## Abstract

The long-term mortality risk of natural disasters is a key threat to disaster resilience improvement, yet an authoritative certification and a reliable surveillance system are, unfortunately, yet to be established in many countries. This study aimed to clarify the mechanism of post-disaster indirect deaths in Japan, to improve the existing disaster recovery evaluation system and support decision making in public policy. This study first investigated the definition of indirect deaths via a literature review before examining the observed number of indirect deaths via case study, census data from the Population Demographic and Household Surveys, other social surveys, and reports in the case of the Great East Japan Earthquake and Tsunami, which severely damaged northeastern Japan, especially the three prefectures, which are the target areas in this context (i.e., Fukushima, Iwate, and Miyagi). It was found that the reported number of indirect deaths was significantly underestimated. In total, 4657 indirect deaths were estimated to have occurred in the target prefectures. This was higher than the reported number, which was 3784. The overall statistics established via collaboration between local administrations and governments can be improved to provide better reference for researchers and policymakers to investigate the long-term effects of natural disaster.

## 1. Introduction

The Sendai Framework for Disaster Risk Reduction 2015–2030 was adopted at the Third United Nations World Conference on Disaster Risk Reduction during 2015 in Sendai, Japan, which includes four priorities and seven targets for the world [1,2]. Target A was to reduce global disaster mortality by 2030, for which the establishment of a mechanism and a mortality database was advocated [2]. The common classification of disaster mortality consists of direct and indirect deaths. Yet, it is still unclear how to count indirect death due to limited surveillance periods, as well as inappropriate definitions and criteria [3]. In fact, indirect death counting is still completely overlooked in some studies [4], as indirect loss data are generally not available [5]. To improve the utility of the Sendai Framework for Disaster Risk Reduction, it is necessary to address the issues of data collection and monitoring through identification and collective consideration, which holistically cope with the complexities associated with defining, reporting, and interpreting disaster mortality data regarding different challenges for different types of hazard events and subsequent disasters for this target [6].

A study of disaster-related deaths caused by Hurricane Andrew published in 1996 initially included indirectly related deaths as those resulting from any other disaster-related event, such as evacuation or cleanup [7]. In 2017, the Center for Disease Control and Prevention (CDC) defined it as any deaths that occur when unsafe or unhealthy conditions are present during any phase of a disaster (e.g., pre-event or preparing for the disaster, during the disaster event, or post-event during cleanup after a disaster) [8]. Although it is named disaster-related death (SaiGaiKanRenShi) in Japan, it is officially defined as death due to injuries aggravated by disaster hazards or illness caused by physical burden during evacuation, and it is recognized as such by the Law of Condolence Money (Act No. 82 of 1973) [9], which also recognizes such deaths that occur post disaster the same way [10,11].

**Figure 1:**
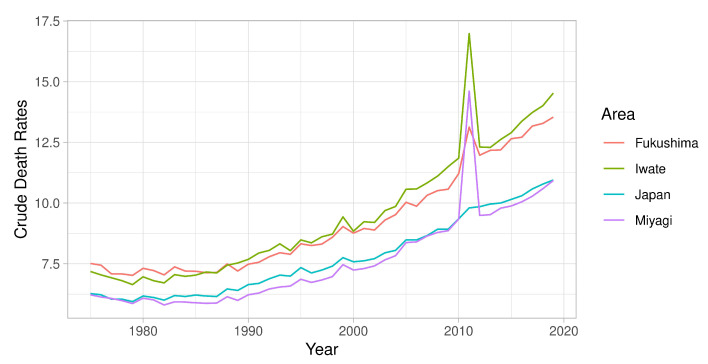
Annual all-cause crude death rates per 100,000 persons in Fukushima, Iwate, Miyagi, and Japan from 1975–2019.

The case of this study is the Great East Japan Earthquake and Tsunami (GEJE) in 2011 that triggered a tsunami and the Fukushima nuclear accident, which caused a total of 19,759 direct deaths [12] and 3784 reported indirect deaths [13]. This resulted in an extreme spike in annual crude death rate that has slowly progressed since 1987 (Figure 1) in the three targeted prefectures studied in this context, along with the general situation of Japan. Compared to direct deaths, indirect deaths are too subtle and intrinsic to be clearly seen or monitored. In Japan, the Reconstruction Agency publishes indirect official deaths based on analysis of death certifications, selection of condolence applications from municipal authorities, and hearings with government officers [14]. Although this surveillance system has been criticized for its biased screening process [15], they have still been monitoring indirect deaths resulting from natural disasters since 1995 and providing the country with authoritative statistics on the extent of the long-term effects of natural disasters.

To clarify the mechanism of post-disaster indirect deaths and assess Japan’s surveillance system, a wide range of literature is reviewed to compare its definitions, and an excess mortality model was built to estimate the death toll of indirect mortality. Excess mortality is an epidemiological word defined as mortality above what would be expected based on the noncrisis mortality rate in the population of interest [16] and is calculated by subtracting the expected number of deaths from the observed number of deaths [17]. It is considered a better indicator for monitoring the scale of a pandemic such as COVID-19 [18], as well as investigating the long-term effects of natural disasters. After investigating 103 countries and territories, we found that in several of the worst-affected countries(Peru, Ecuador, Bolivia, Mexico) the excess mortality was above 50% of the expected annual mortality [19]. The pandemic not only caused internal and external tension in different countries [20], but also can be related to the dismal state of people’s wellbeing due to the unplanned lockdown and subsequent period of socio-economic and health crisis [21], which is likely to contribute to indirect death. In this paper, the added value is that, more so than for direct death, the number of indirect deaths that cannot be easily collected is the initial focused in this methodology, which was developed for COVID-19. A constant value of excess death was first used in the field of natural disasters to explore deviations from baseline (expected death) in the case of the mid-July 1995 heat wave in Chicago [22]. In another instance, the temporal trends in the risk of excess mortality were assessed by constructing a Poisson regression using age, city, and year, which showed that indirect deaths that were impacted by physical health problems in Soma City and Minamisōma City were most severe in the first month after the disaster [23].

The hypothesis of this study is that post-disaster indirect mortality has been underestimated among prefectures, cities, towns, and villages. Thus, better accountability for indirect deaths is required so we can monitor the impact of disasters more comprehensively [24]. Using the case of the Great East Japan Earthquake and Tsunami, this study aims to inspect Japan’s indirect post-disaster death surveillance system and clarify a mechanism to optimize its certification, which plays a crucial role in providing reliable statistics for recovery evaluation.

## 2. Data and Methods

This multidisciplinary study originally adopts the epidemiological methodology from a socioeconomic perspective by studying larger demographic data from severely damaged areas to examine the indirect surveillance system in Japan, and aims to provide reference for policymakers and researchers to improve this system in Japan or establish a better one in other countries. This paper is divided into two parts: the first part is a literature review intended to qualitatively analyze and investigate indirect deaths, as well as the pros and cons of the existing surveillance system; the second part uses a machine learning model to quantitatively analyze the gap between the expected and observed indirect death toll.

To firmly define indirect death, government reports from the Reconstruct Agency and Cabinet Office and research articles from the field of epidemiology, social science, and public policy are reviewed, in which the researchers performed their studies from selection of appropriate data to conducting analytic work and forming the concept. Regarding data selection, Japan’s research related to indirect death focus mainly on domestic data, from central departments such as the Cabinet Office and local governments in disaster-prone areas such as Kobe [25], while global researchers turn to a wider range of information sources, including institutions such as the CDC. With regard to disasters, it is generally covered because among limited literature directly related to indirect death, some focus on storm hazards, some study seismic disasters, and others impose interests of all kinds. For example, government reports such as those from the Cabinet Office [9] and CDC applied their indirect death to all disasters globally, while Loris and Ueda kept an eye on the local area. Regarding the affected population, most of those studies were carried out in high-frequency areas where geological movements can cause more deaths that sometimes are thousands of people than any other type, costing dozens of lives. In terms of the time frame, it varies from study to study.

Since 2020, excess mortality has become a popular buzzword because it is considered the most objective indicator of the COVID-19 death toll [26,27]. Under the circumstances of the pandemic, officially reported deaths and demographic data are commonly used to calculate excess mortality. However, when it comes to natural disasters such as hurricanes and tsunamis, although the amount of death certifications and vital registration mortality data that can be utilized, respectively, to obtain observed deaths is limited, it is an ingenious and innovative way to predict death tolls by coping with the uneasy situation of confirming the true number of indirect deaths due to the lack of directly observed indirect deaths [19,28,29].

### 2.1. Data and Tools

Twenty-four official reports associated with the indirect deaths caused by the GEJE published every June and December from 2011 to 2021 by the Japan Reconstruction Agency were scrutinized, from which the number of observed indirect deaths was acquired. The observed number of direct deaths was obtained from the latest report published in 2021 by the Fire and Disaster Management Agency. The number of the registered population and all-cause deaths in 131 municipalities from 2000 to 2021 was extracted from resident registrations that are included in the Population, Demographic and Household Surveys based on the Basic Resident Ledger published every August by the Ministry of Internal Affairs and Communications on the e-Stat Portal Site of Official Statistics of Japan. Since 2000, many municipalities have been merged into one (e.g., Yamagata village was combined into Kuji city in 2006). In this study, all the data from the despaired municipalities are added and counted into the latest merged municipalities. Moreover, geographic data of Geo JSON was obtained from the Ministry of Land, Infrastructure, Transport and Tourism of Japan. Although it is known from interviews that the Ministry of Health, Labor, and Welfare of Japan manages a database of death certifications, our requests for access to death certifications were rejected by both the local and national government. Thus, all the data used in this context are open access and available from official government websites. As for tools applied in this analysis, Python was used for geographical analysis and data cleaning by means of Jupyter Notebook and Google Colaboratory, while R was used for data organization, regression analysis, and plot export, using RStudio and RStudio Cloud.

### 2.2. Method

The mega disaster can be regarded as a colossal natural experiment [30,31], in which the control group (all municipalities) turned into an experimental group from 11 March 2011 due to the intervention of the GEJE. Morita’s research quantified the excess mortality of two cities in Fukushima from 2015 to 2016 [23], while Uchimura estimated that the mortality ratio in the month post disaster, comparing the three prefectures with other prefectures was at 1.20 for those aged 60–69 years old [32]. Nevertheless, as an essential indicator of long-term environmental and health effects, the number of indirect deaths is still unknown [33]. Regarding almost all prefectures in Japan, the total reported indirect deaths on 31 March in 2019 reached 99.9% of the accumulated total reported indirect deaths in 2020, and dropped to zero and stopped growing after 31 March in 2020. The first death attributed to the COVID-19 pandemic in Japan occurred on 10 February 2020 [34], when it began to affect all-cause mortality and population. Thus, although we intended to observe the period 2000–2020, 2019 is selected as an end node for the long-term effects of GEJE. Population and all-cause death between 2000–2010 and 2018–2019 are deemed as the control group, while those occurring between 2011–2017 are deemed the experimental group. In order to create a control group for 2011–2017, data between 2000–2010 and 2018–2019 were used to train a Gaussian process model Equation (1) to estimate a set of plausible coefficients for calculating excess deaths in Equation (2). Then, Equation (3) was used to collect indirect deaths from different cohorts.

(1) $$N_{m,y}=\alpha_{m}\cdot Y_{\left(2000-2010\&2018-2019\right)}+\beta_{m}+\epsilon ,\;\;\epsilon ∼N\left(0,\sigma^{2}\;\right)$$

Regressing the Gaussian process with a Bayesian approach functions well on small time-series data sets capable of providing uncertainty measurements on the predictions [27] for each municipality employed as the smallest unit. In the generalized linear model Equation (1), *N* denotes the observed number of all-cause deaths in the municipality of *m* and the year of *y*. The constant α is a linear slope for the municipality of *m* across years. β refers to a separate intercept as fixed effects in each municipality of *m* from the difference in population structure, migration rate and other socioeconomic factors. *Y* is an independent variable ranging from the years 2000–2010 and 2018–2019, and ϵ is the Gaussian noise following the normal distribution fluctuating around zero.

(2) $$E\left(N_{m,y}\;\right)=\hat{\alpha_{m}}\cdot Y_{2011-2017}+\hat{\beta_{m}}$$

(3) $$I=\sum_{j}^{C}\left\{\sum_{i}^{2011-2017}\left[N_{j,i}-E\left(N_{j,i}\right)\right]-D_{j}\right\}$$

In Equation (1), all α and β estimated from Equation (2) are used to calculate the expected number of counterfactual deaths in municipality *m* in year *Y* (2011–2017). Equation (3) was developed to predict the number of indirect deaths *I* by summing municipality *j* in cohort *C* after adding up the excess numbers of year *i* 2011–2017. By referring to a specific cohort, we can obtain the predicted number of indirect deaths by resetting *C* (e.g., prefectures, municipality, etc.).

## 3. Results

In our preliminary study, it was gauged by baseline death rate between 2010 and 2019 that 624,695 all-cause deaths would have occurred if the GEJE did not happen, compared to the 651,442 observed deaths that did occur according to Vital Statistics during 2010 and 2019. Correspondingly, after deducting 19,759 direct deaths that were investigated by the Fire and Disaster Management Agency from the 26,747 excess deaths, the number of indirect deaths should be 6988, which means approximately 3204 deaths were undercounted by the surveillance system.

### Literature Review

In this session, government reports and research articles are reviewed in fields such as epidemiology, public policy, and social science to study indirect death and the surveillance system. We investigated different definitions and causes of indirect death as a background study and clarified the history, mechanism, advantages, and disadvantages of the surveillance system.

In Japan, the recognition of indirect deaths is closely related to the condolence policy following Act No. 82, launched in September 1973, which initially regulated condolence payments of up to JPY 500,000 to the immediate family of the victim who perished directly or indirectly from natural disasters [35]. After several rounds of legal amendments, the payment has been increased to up to JPY five million since 1991. When the concept of indirect death (Saigai Kanrenshi in Japanese) was first introduced in January 1995 (Table 1) when the Great Hanshin-Awaji Earthquake struck west Japan, 14.3% of 6405 total casualties were classified as indirect deaths [36,37].

**Figure 2:**
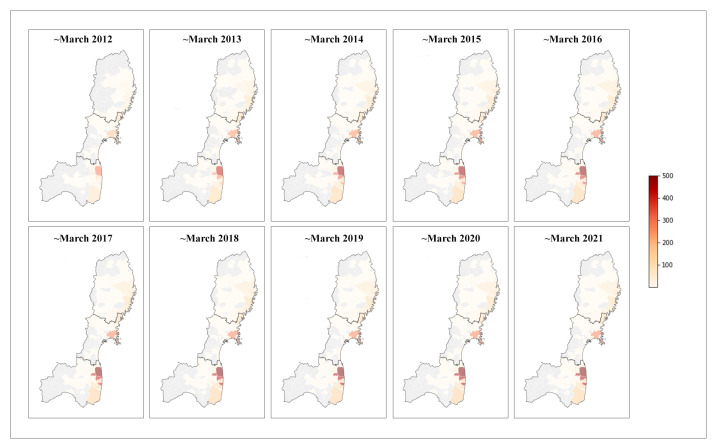
Annual geographical distribution of accumulated reported indirect death number of the Great East Japan Earthquake and Tsunami of 131 municipalities in Miyagi, Fukushima, and Iwate from March 2012–2021.

Based on the definitions in Table 1, indirect death is studied and described in various ways by classifications and causes. Two weeks are conditionally implied as a key node for indirect deaths despite the dissimilarity in the type and scale of disasters, regions, or emergency management. Ueda demonstrated a watershed of two weeks (up to three weeks in the case of a mega disaster such as GEJE) between the chronic phase and the acute phase, which can be further differentiated as the hyper-acute phase, acute phase, and sub-acute phase [25,40,41]. Since the elderly are commonly regarded as a vulnerable group, Ehren pointed out that 50% of those who died from cardiovascular causes post-disaster usually died within two weeks [44]. In Loris’ study, two weeks is a general borderline to determine immediacy [3]. Since the end node of disaster effects depends on the disaster scale to a great extent, in GEJE the reported number of indirect deaths almost stopped increasing at 469 in 2018 for Iwate, 929 in 2020 for Miyagi, and 2,314 in 2020 for Fukushima [13], in which Sendai city is demonstrated by five wards (i.e., Aobaku, Izumiku, Miyaginoku, Taihakuku, and Wakabayashiku) (Figure 2).

Thanks to infection control following GEJE [46], infectious illness is not a major cause of indirect death. The four leading causes comprise pneumonia, coronary heart disease, stroke, and cancer [23]. Among cities and towns hit by tsunami, a higher percentage of flooded households was associated with a higher risk of indirect death, lower expenditures on outpatient medical care, and lower expenditures on home care services [32]. It was reported that 50% of indirect death on average was due to physical and mental exhaustion of evacuating and living in a shelter, of which 60% was attributed to evacuees from Fukushima [14]. In the case of the 2004 Indian Ocean tsunami, indirect mortality was positively correlated with poor post-tsunami psychosocial health for males and loss of spouse for females [47].

To be recognized as a case of indirect death for individuals in Japan, someone (e.g., immediate family members) must submit a list of materials to the administrative authority or local government for application. After the screening process via the Disaster Condolence Grants Committee (consists of lawyers, doctors, administrative officers, and other professionals who are coordinated by the municipal/prefecture-level government), recognition and aid are then granted to the applicant. Since the amendment in 1991, if the victim is the breadwinner of the applicant’s family, JPY 5 million is awarded. If the victim is not, JPY 2.5 million yen is awarded instead. Accordingly, one-half of the payment is covered by the national government, one-fourth by the prefecture government, and one-fourth from local government of the municipality [48,49,50]. After reviewing reports and interviewing the government staff, it is clarified that the detailed reports from the Reconstruction Agency have been published twice a year since 2011 through continuous collection and analysis of data from death certifications, committee reports, and hearing from professionals and local governors, etc. [14]. Successful applications for condolence payments are consequently counted by committee reports and death certifications. Indirect deaths with no applications are only considered by scrutinizing other reports to a certain degree.

**Figure 3:**
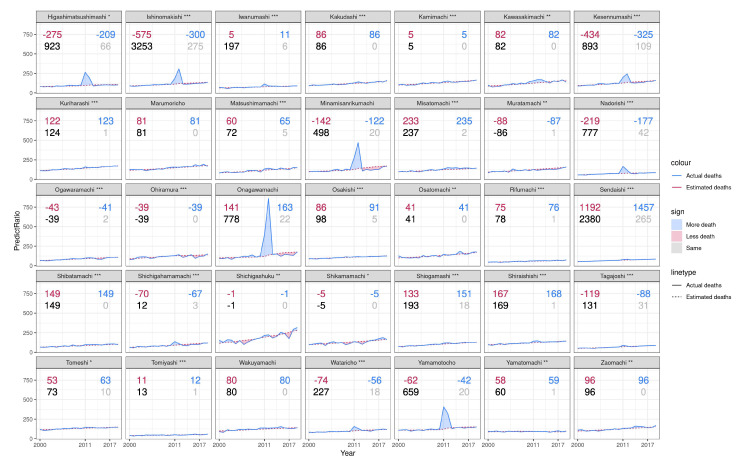
All-cause death per 10,000 population of the municipality in Miyagi during 2000 and 2020. The bottom right gray number of each plot is the number of observed indirect deaths by March 2019 reported by the Reconstruction Agency; the bottom left black number of each plot is the estimated number of total excess deaths in the prefecture between January 2011 and December 2017. The top right blue number of each plot is the estimated indirect deaths calculated by subtracting reported direct deaths from total excess deaths; the top left red number of each plot is the under-counted number calculated by subtracting reported indirect deaths from estimated indirect deaths. * = *p* < 0.05, ** = *p* < 0.001, *** = *p* < 0.001.

In Miyagi (Figure 3), it was observed that indirect deaths are under-reported in some municipalities, including Onagawamachi and Shiogamashi (in Japanese, the suffix of machi or cho refers to a town as a political district; shi refers to the city and mura or son refer to the village), while they were over-reported in municipalities such as Higashinatsushimashi and Kesennumashi etc. The rising rate in Sendaishi is stable, with a higher number of indirect deaths, due to its larger and younger population. Furthermore, the population in rural areas inclusive of Shichigashuku, Shikamamachi and Ohiramura was found to fluctuate dramatically due to the disaster’s long-term effects.

**Figure 4:**
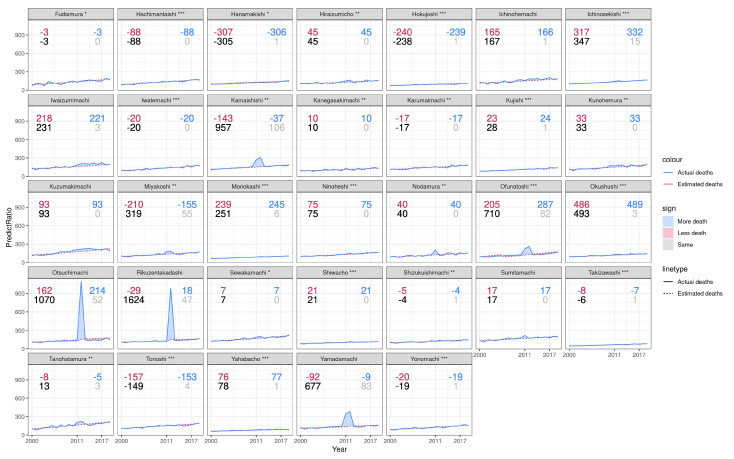
All-cause death per 10,000 population of municipality in Iwate during 2000 and 2020. The bottom right gray number of each plot is the number of observed indirect deaths by March 2019 reported by the Reconstruction Agency. The bottom left black number of each plot is the estimated number of total excess deaths in the prefecture between January 2011 and December 2017. The top right blue number of each plot is the estimated indirect deaths calculated by subtracting reported direct deaths from total excess deaths. The top left red number of each plot is the under-counted number calculated by subtracting reported indirect deaths from estimated indirect deaths. * = *p* < 0.05, ** = *p* < 0.001, *** = *p* < 0.001.

In Iwate, indirect deaths are under-reported in municipalities including Ichinosekishi, Ichinohemachi, Ofunatoshi, and Okushushi, while they were over-reported in municipalities such as Minamisanrikumachi and Wtaricho (Figure 4). In some municipalities, the estimated number of indirect deaths turned out negative because excess mortality was lower than expected due to mortality selection that dominated scarring effects [47].

**Figure 5:**
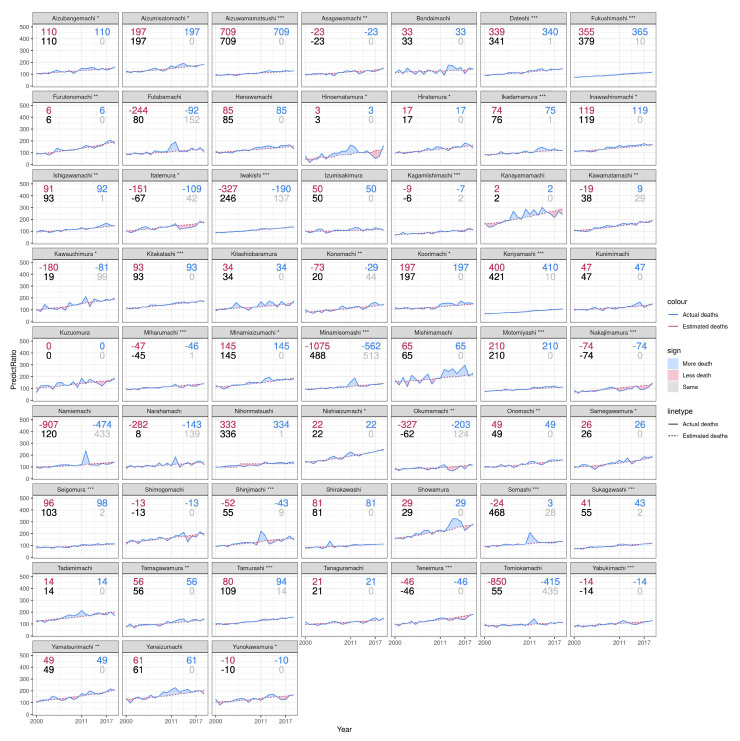
All-cause death per 10,000 population of municipality in Fukushima during 2000 and 2020. The bottom right gray number of each plot is the number of observed indirect deaths by March 2019 reported by the Reconstruction Agency; the bottom left black number of each plot is the estimated number of total excess deaths in the prefecture between January 2011 and December 2017. The top right blue number of each plot is the estimated indirect deaths calculated by subtracting reported direct deaths from total excess deaths; the top left red number of each plot is the undercounted number calculated by subtracting reported indirect deaths from estimated indirect deaths. * = *p* < 0.05, ** = *p* < 0.001, *** = *p* < 0.001.

In Fukushima (Figure 5), where the nuclear disaster occurred at the Daiichi Nuclear Power Plant in Okumamachi, the number of displaced evacuees peaked at 165,000 in May 2012 and subsequently decreased to 37,000 in January 2021 [51]. Our analysis indicates that the mortality pattern in municipalities such as Showamura, Koorimachi and Shimogomachi destabilized after 2011. Indirect deaths in municipalities including Hanawamachi, Koorimachi, and Motomiyashi were under-reported, while they were over-monitored in municipalities such as Kawamuchimura and Narahamachi.

## 4. Discussion

The impact of the megadisaster GEJE on the population was more profound than that reported by official statistics or news. A total of 4657 indirect deaths were estimated in Fukushima, Iwate, and Miyagi prefectures, which is much higher than the reported number 3784, demonstrating a possible underestimation of indirect deaths in the case of the GEJE. To predict indirect deaths with more precision, all municipalities in the three prefectures were used as the smallest unit in the demographic panel data.

**Figure 6:**
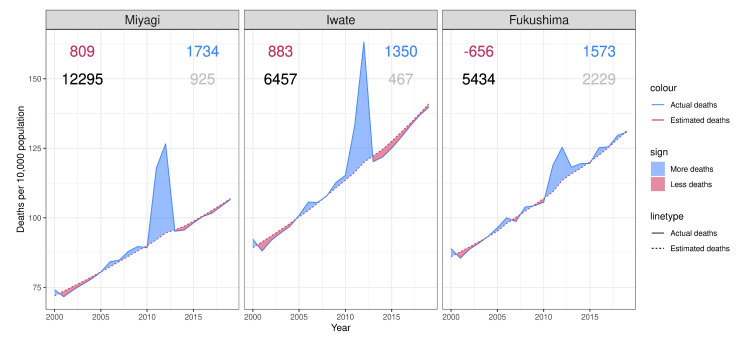
Actuality and estimation of all-cause crude death rate during 2000–2019 in three prefectures The bottom right gray number of each plot is the number of observed indirect deaths by March 2019 reported by the Reconstruction Agency. The bottom left black number of each plot is the estimated number of total excess deaths in the prefecture between January 2011 and December 2017. The top right blue numbers of each plot is estimated indirect death calculated by subtracting reported direct deaths from total excess deaths. The top left red number of each plot is the under-counted number calculated by subtracting observed deaths from estimated indirect deaths.

In Miyagi, it was estimated that 12,295 excess deaths occurred between January and December 2017, with 1734 possible indirect deaths. Subtracting the 925 reported direct deaths, we can confirm that 809 cases were ignored. In Iwate, 6457 excess deaths were captured by the model, which predicted 1350 indirect deaths compared to the 467 reported indirect deaths. Consequently, 883 cases were missed by the surveillance system. In Fukushima, the predicted 1573 indirect deaths fell below the reported 2229 indirect deaths, which means that 656 cases were overreported. By Figure 6, mortality selection can be identified on the plots of Miyagi and Iwate, but in Fukushima, the scarring effects slightly dominated the mortality selection.

The concept of mortality selection originated from Darwin’s theory of natural selection [52] and was later developed by Endler, who demonstrated it as a restatement of ’survival of the fittest’ tautology [53]. Natural selection can be divided into sexual and non-sexual selection in a narrower sense; in the latter case, mortality selection and fecundity selection are included [53]. Mortality selection can be defined as a phenomenon where members with disadvantageous characteristics are more likely to die as the cohorts age [54,55]. Another similar term, selective mortality, is defined as a process in which disadvantaged individuals die at a younger age than their more advantaged peers [56]. It can be used to explain the drop in actual all-cause deaths after 2011 below the baseline death rate, because disadvantaged cohorts that are vulnerable in terms of physical, mental, and socioeconomic status in the targeted area died before when they would usually have died. On the contrary, scarring effects, which can be defined as a series of relatively negative effects on the post-disaster population, could lead to early death during their lifetime, as childhood adversities could lead to adult psychiatric disorders and eventually suicide [57]. Scarring effects are also regarded as the effects of a persistent change in beliefs about the probability of an extreme and negative shock [58]. Under several circumstances, the all-cause death rate can return to the baseline level plausibly, but only if there is a balance between mortality selection and scarring effects post the natural disaster. If the balance is not reached, the actual death rate fluctuates.

In the case of the GEJE that occurred on 11 March 2011, 17.0% out of 22,199 total post-disaster casualties [59,60] were classified as indirect deaths, signaling a worrying increase and a need to reconsider the underlying reasons as well as the effectiveness of disaster recovery, per se. Indirect death is a chronic and unignorable problem that can also be seen in the case of other natural disasters. For example, a review of 59 US tropical cyclones showed that the number of indirect deaths was just as large as the number of direct deaths, in which the main causes turned out to be cardiovascular failure, vehicle accident, evacuation failure, and power failure [61]. Moreover, underestimation occasionally happens in other cases of disasters as well, as demonstrated by related research articles. For instance, in the case of Hurricane Maria, it is estimated that there were 2975 excess deaths, in contrast with the sixty-seven deaths primitively reported by the Puerto Rico Department of Health [62].

Through unstructured interviews with residents of Ishinomaki and Sendai cities, six interviewees who lived in different places stated that they have great empathy for the suffering and death of their relatives or friends, but are not sure about condolence money or the concept of indirect death. It can be seen that their trauma from the past is connected to the notions of empathy and also to mental instability, which was reported to be one of the reasons for 13 suicides among the number of indirect deaths [14,63]. Understanding the mechanism of indirect death should take place from a complex system perspective in which indirect death correlates with social–cultural, natural, and physical environments [64].

The limitations of the study are the precision of the model and the volume of data, which can be improved by refining the model and expanding the dataset through further study. To provide more comprehensive evidence, more multidisciplinary studies are needed to explore causal relationships between socioeconomic effects and indirect death, as well as case studies investigating those municipalities where estimated indirect deaths significantly deviated from observed indirect deaths. Beyond improving the existing surveillance system for the future, overlooked deaths must also be counted with a more comprehensive methodology that considers migration and other variables. For greater generalization, more comparisons of different disasters should also be investigated.

## 5. Conclusions

The study estimated excess mortality in three prefectures severely damaged during the Great East Japan Earthquake and Tsunami. In total, 873 deaths were found to have not been reported as indirect deaths. Our methodology and results can be used as evidence to demonstrate that optimizing the existing surveillance system by improving death certification and standardization can provide better statistics in terms of disaster recovery evaluation for researchers and policy makers.

This paper makes a two-fold contribution to policy recommendations against the imperfections that exist in policies of the Japan condolence law and the Sendai framework. In total, 279 items of news reported by searching both keywords of indirect death and the Great East Japan Earthquake and Tsunami can be found via Google news by setting the time range between 11 March 2021 and 11 March 2022, which implies that indirect deaths caused by the GEJE are still a problem that receives attention from Japan society even after 10 years. However, how the number of indirect deaths in Japan is collected remains problematic, especially the immoderate reliance on condolence law, which cannot cover the perished who do not have any relative or spouse to apply for the condolence subsidy. Therefore, this study pointed out that there should be more policies to cooperate with the condolence law to maximize the accuracy of the statistics. On the other hand, to follow the status of whether and how the target A in the Sendai framework is being achieved, the number of deaths and missing persons attributed to disasters per 100,000 population of each country is collected by the Disaster Loss Data Collection Tool (called “DesInventar Sendai”), which is a subsystem of the Sendai Framework online monitoring tool. Despite the mention of attribution, death is applicably defined as the number of people who died during the disaster or directly after, as a direct result of the hazardous event in the Technical Guidance for monitoring and reporting on Progress in achieving the global targets of the Sendai Framework for Disaster Risk Reduction. This study addresses the importance of indirect death that can also be attributed to disasters and demonstrates the direction for developing a better methodology to monitor the number of deaths.

This study not only implies that a surveillance system monitoring long-term effects, such as the statistics of indirect death caused by natural disasters, can be built through methods inclusive of the existing policy and developing related policy, but also indicates the importance of upgrading the Data Collection tool serving the Sendai Framework by improving the criteria used to monitor countries’ data and involving the number of indirect death that can also be attributed to disasters. In addition, social factors such as social networks that can underpin apparently spontaneous actions [65] should also be investigated to further develop the model in this study. Global demographic data can be collected to feed the model, which can be used more precisely to verify the exactness of the reported death toll or to estimate the number of indirect deaths in countries where the statistics for natural disasters are not yet well developed.

## Figures and Tables

**Table 1:** Literature review of definition of indirect death.

Reference	Terminology	Time frame	Definition/Description
Cabinet Office (2020) [9]	Disaster-related death	No implication	Death due to injuries aggravated by disaster hazards or illness caused by physical burden during evacuation
Loris et al. (2007) [3]	Indirect Death	Two weeks	Death resulting from suicide; fatal injury occurring during clean-up; post-disaster pulmonary embolism on account of sheltering in motor vehicles.
	Disaster-Triggered Deaths	Two weeks; one year	Death resulting from disruption of care for post-disaster chronic illness; suicide.
Asim et al. (2006) [38]; NWS/NOAA (2005) [39]	Indirect Death	No implication	Death that occurs in the vicinity of a hydro-meteorological event, or after it had ended, but were not directly caused by impact or debris from the event.
Ueda et al.(1996) [25]; Ueda (2014) [40]; Ueda (2016) [41]	Post-disaster-related death	72 h; two or three weeks depends on the scale; three months	Deaths due to indirect causes such as psychological shock and severe evacuation conditions, even if the disaster did not cause a wound.
Debra (1999) [7]; CDC (2017) [42]	Indirectly related disaster death	Any phase	Death that occurs when the unsafe or unhealthy conditions present during any phase of the disaster (i.e., pre-event, during the actual occurrence, or post-event).
Nagaoka (2004); MHLW (2011); Miyamoto (2013) [43]	Disaster-related death	One month; six months	Death that occurs due to abrupt change of environment or suicide caused by mental illness or stress from the disaster during six months post disaster.
Ehren B. (2001) [44]	Indirect death	Approximately two weeks following the event.	Deaths not primarily resulting from the initial and physical impact the hurricane.
Nishant et al. (2018) [45]	Indirect death	No implication	Deaths resulting from worsening of chronic conditions or from delayed medical treatments that may not be captured on death certificates.
Hyogo Prefecture, MHLW (1995) [36]	Disaster-related death	No implication	Deaths certified by the Disaster Condolence Grants Committee with a reasonable causal relationship to the earthquake disaster.

## Data Availability

Please contact the corresponding author for the information of data availability.

## Abbreviations

The following abbreviations are used in this manuscript:

CDC	Center for Disease Control and Prevention.
GEJE	Great East Japan Earthquake and Tsunami.
MHLW	Ministry of Health, Labour and Welfare of Japan.
NOAA	National Oceanic and Atmospheric Administration.
NWS	National Weather Service.
UNDRR	United Nations Office for Disaster Risk Reduction.
WHO	World Health Organization.
